# Study protocol: DEcisions in health Care to Introduce or Diffuse innovations using Evidence (DECIDE)

**DOI:** 10.1186/s13012-016-0412-8

**Published:** 2016-04-05

**Authors:** Simon Turner, Stephen Morris, Jessica Sheringham, Emma Hudson, Naomi J. Fulop

**Affiliations:** Department of Applied Health Research, University College London, 1-19 Torrington Place, London, WC1E 7HB UK

**Keywords:** Cancer, Decision-making, Discrete choice experiment, Ethnography, Evidence, Innovation, Ophthalmology, Qualitative, Service improvement, Stroke

## Abstract

**Background:**

A range of evidence informs healthcare decision-making, from formal research findings to ‘soft intelligence’ or local data, as well as practical experience or tacit knowledge. However, cultural and organisational factors often prevent the translation of such evidence into practice. Using a multi-level framework, this project will analyse how interactions between the evidence available and processes at the micro (individual/group) and meso (organisational/system) levels influence decisions to introduce or diffuse innovations in acute and primary care within the National Health Service in the UK.

**Methods/design:**

This study will use a mixed methods design, combining qualitative and quantitative methods, and involves four interdependent work streams: (1) rapid evidence synthesis of relevant literature with stakeholder feedback; (2) in-depth case studies of ‘real-world’ decision-making in acute and primary care; (3) a national survey and discrete choice experiment; and (4) development of guidance for decision-makers and evaluators to support the use of evidence in decision-making.

**Discussion:**

This study will enhance the understanding of decision-makers’ use of diverse forms of evidence. The findings will provide insights into how and why some evidence does inform decisions to introduce healthcare innovations, and why barriers persist in other cases. It will also quantify decision-makers’ preferences, including the ‘tipping point’ of evidence needed to shift stakeholders’ views. Practical guidance will be shared with healthcare decision-makers and evaluators on uses of evidence to enable the introduction and diffusion of innovation.

## Background

A range of evidence informs healthcare decision-making, from formal research findings [1] to ‘soft intelligence’ or local data [2], as well as practical experience or tacit knowledge [3]. However, cultural and organisational factors often prevent the translation of such evidence into practice [4]. As well as the perceived quality of the evidence (e.g. due to its source) and ‘strength’ (as a working definition, the effect or impact shown on health, costs, and patient satisfaction), decisions to implement innovations or improvements in health services are influenced by contextual processes at the micro (individual/group), meso (organisational/system), and macro (regulatory/policy) levels [5]. Using a multi-level framework, this study will analyse how interactions between the available evidence and processes at the micro and meso levels influence decisions to introduce or diffuse innovations. We adopt a process-based approach [6] to the study of evidence use, defining ‘use’ as the ways in which different stakeholders and organisations interact with evidence over time during decision-making processes.

A preliminary, scoping review of the health services research literature on evidence use in decision-making concerning innovation suggests a need for new research in three areas. First, at the micro (individual/group) level, there is a need to determine the combinations of evidence, including practical or local evidence [7], used by a range of stakeholders in decision-making, including different professions [8] and functions or roles [9]. At the individual level, some studies suggest that research evidence plays a lesser role in decision-making relative to other information [10], such as data on local needs [7]. At the group level, access to and preferences for evidence vary by professional group, e.g. hospital staff’s professional background [8], while service payers (commissioners) appear to value practical evidence [9]. How evidence is presented also influences its use [11]. Furthermore, evidence is itself constructed through professional practice, in which different interests, framing of the problem, and personal experience and anecdote all play a role in establishing its relevance and credibility [12]. Thus, further research is needed at the micro level to determine how different stakeholder groups, in different contexts, use evidence to inform decision-making on innovation, including their responses to different forms of evidence, and how potential tensions between codified research outputs, practical evidence (e.g. local audit data), and personal experience or tacit knowledge [13], are reconciled as different forms of evidence are combined in decision-making.

Second, at the meso (organisational/system) level, there is a need to examine how evidence informs ‘real-world’ decision-making processes through in-depth case studies [8], taking into account organisational processes for sharing knowledge [14] and other contextual factors, including strategic fit with local priorities, financial sustainability, and public opinion [11]. Organisational processes influence how evidence is acquired, shared, and applied to inform decision-making. For instance, implementation of national clinical guidance by National Health Service (NHS) Trusts was found to involve senior engagement, clear organisational processes, and use of committees and hierarchies, resources, and information systems [14]. Equally, weak processes for transferring knowledge may act as barriers to its use in decision-making [11]; however, a variety of ‘agencies’ at the meso and macro levels may support the transfer or mobilisation of knowledge [15]. Thus, further research is needed at the meso level to understand how organisational processes, including the local system or context in which decisions are being made, influence the use and interpretation of evidence, including health professionals’ responses.

The inclusion of both micro and meso level processes reflects the theorised interaction between levels in quality improvement processes [5], i.e. organisational and other contextual processes may shape professional responses to evidence, while health professionals’ responses may influence the adoption of innovation in particular contexts.

Third, studies have shown that stakeholders prefer different types of evidence (including quality and strength) [8, 9], but little is known about the strength of these preferences, the potential trade-offs between these attributes in relation to different types of innovation, and how preferences vary by type of decision-maker. In addition, little is known about how other characteristics of evidence and other contextual factors inform decisions to introduce or diffuse innovations. Further research is needed to evaluate these preferences, which we will investigate using a discrete choice experiment.

### Research question and objectives

To address the gaps identified in the current literature, this study will answer the following research question: what is the role of evidence in decision-making on the introduction and diffusion of service innovations in acute and primary care? In order to address this research question, the following objectives for the study have been defined:

1. To identify, using a literature review and stakeholder feedback, the factors that influence the use of evidence in decision-making on the introduction and diffusion of innovations in health care;
2. To assess the use of evidence in informing decision-making on the introduction and diffusion of innovation using ‘real-world’ case studies in acute and primary care;
3. To establish decision-makers’ preferences for evidence (types of evidence, quality of evidence, strength of evidence) to inform the introduction and diffusion of innovations;
4. To develop guidance for decision-makers and evaluators to support the evaluation and application of evidence to enable innovation.

## Methods/design

This multidisciplinary study uniquely brings together different methodological and disciplinary perspectives (ethnography, organisation studies, improvement science, health economics) to study the role of evidence in decision-making with the aim of meeting these objectives. The following work streams, which are interdependent and will inform one another, are planned in relation to each objective. Methods for data collection and analysis are described by work stream.

### Work stream 1: rapid literature review and stakeholder feedback on evidence use

The rapid review, with stakeholder feedback, has two purposes: (a) to map the types of information used to inform decision-making in different contexts and (b) to identify factors at the micro and meso levels that influence how this information informs decision-making on innovation. We will then obtain stakeholder feedback on the compiled evidence in relation to these purposes using focus groups to identify any gaps or themes that need to be developed further.

The literature review will identify examples of evidence use in decisions to adopt innovation from studies conducted on the NHS and health systems internationally. To identify papers, relevant social science and biomedical databases will be searched, including PubMed, ISI Web of Knowledge, and Business Source Complete, using search terms in the title or abstract. Search terms, that may be combined, will include the following: ‘decision-making’, ‘service innovation’, ‘evidence use’, ‘innovation adoption or diffusion’, ‘professional roles’, and ‘organisational factors or processes’. A hand search of selected management and health policy journals and books will be undertaken; bibliographies of recent and highly relevant papers will also be consulted. Studies of decision-making at the national health system level and in public health will be excluded. The review will be based predominantly on primary qualitative studies and systematic and other types of review. To map types of information used in decision-making, we will include quantitative studies that provide descriptive statistics on evidence use, as appropriate.

Through thematic analysis of the compiled literature [16], we will develop a conceptual framework that identifies the (a) types of evidence used to inform decision-making in different contexts and (b) professional (micro) and organisational (meso) processes that shape how evidence is used to inform innovation adoption. The framework will include decision-making in different settings (acute and primary care) and describe these processes from different stakeholder perspectives (service providers, commissioners, and patient representatives). To test and develop the thematic analysis further, we will then obtain stakeholder feedback on the conceptual framework using focus groups to identify any gaps or themes that require further exploration. The focus groups will also be used to determine how stakeholders define ‘acceptability’, ‘credibility’, and ‘strength’ in relation to different types of evidence and in relation to different decision-making contexts.

The views of four groups of stakeholders will be obtained through the focus groups: (1) acute care providers, (2) primary care providers, (3) service commissioners, and (4) patient representatives. Four focus groups, with 8–10 participants in each group, will be structured using discussion points derived from the rapid literature review. Telephone interviews will be used for participants not able to attend a face-to-face focus group. National participants will be identified using different channels. Through the National Institute of Health Research Collaborations for Leadership in Applied Health Research and Care (CLAHRC) North Thames, which has 54 partners including hospitals, local authorities, and commissioners, we have links across the 13 CLAHRCs nationally which will be used to reach participants.

### Work stream 2: in-depth case studies

In-depth case studies will be conducted on the use of evidence in ‘real world’ decision-making concerning the introduction or diffusion of three service innovations in acute and primary care. Case studies were chosen because they allow complex phenomena to be studied in-depth, allowing both the case (here, the use of evidence in decisions to adopt innovations) and the context (professional and organisational processes) to be taken into account, as well as interactions between the two [17]. This approach also addresses a need for ethnographic methods to enable direct observation of ‘live’ decision-making processes [8].

#### Sampling framework for case studies

As shown in Table 1, the sampling framework for the three case studies covers different settings (acute and primary), innovation stages (new and diffusion), type and strength of evidence (academic research, national guidance, and local pilot data), and organisational contexts (including different approaches to the implementation of innovation). The case studies complement each other in showing how the use of evidence to inform decision-making varies across different care settings, among different types of decision-maker, stages of innovation, and types of evidence (including perceived strength). For each case study, guiding questions aim to capture the influence of processes at the micro (individual/group) and meso (organisational/system) levels on the use of evidence to inform decision-making on innovation (set out below). Lessons from the case studies will be brought together, with quantitative data from the national survey and discrete choice experiment, to show how interactions between evidence use and factors at the micro and meso levels create a ‘tipping point’ for innovation in different contexts. Suggestions will also be made for improving evidence use to support innovation in less receptive settings.

**Table 1:** Sampling framework for case studies

Innovation case study	Setting	Innovation stage	Evidence	Context
1. Reconfiguring stroke services	Acute; Greater Manchester (GM) and other areas reviewing services	Diffusion	‘Strong’; research shows improvements in mortality in London [18, 19]	Major system change; involves multiple providers and commissioners
2. New national guidance on referral for suspected cancer	Primary care; GP practices in two local health economies with different mix of actors supporting implementation (clinical networks, third sector)	New	‘Inconclusive’; national guidance lowers referral threshold [22], with the aim of reducing emergency admissions and diagnosing at earlier stage	Top-down change; responses of GPs and actors at local health economy level
3. New virtual clinics within extended network of eye services	Acute/community outreach; clinics across large metropolitan area and surrounding region	Diffusion	‘Weak’; local pilot data suggesting reduced patient journey time [26], but lack of patient outcome data and evidence for networked clinics	Organisational network; from pilot to wider implementation of networked clinics

#### Case study 1: responses to evidence on reconfiguring stroke services

Evidence produced by members of the research team has shown that centralising stroke services to create a smaller number of high-volume units in London has improved patient outcomes [18, 19]. This evidence has in part influenced a decision to further centralise stroke services in Greater Manchester [20]. A number of areas across the UK have also reviewed the configuration of stroke services locally and appear to be responding differently to the evidence. For example, Greater Glasgow and Clyde initially decided not to implement the London model, although a further service review is planned, while in Birmingham and the surrounding area, partial reconfiguration of services has been undertaken, despite support for fuller centralisation [21]. Analysing the differing responses of a number of these areas will allow greater understanding of what is needed for evidence to become a tipping point for change. This case study will focus on the following:

1. At the micro level: how both stroke clinicians and senior managers within individual provider organisations’ use, and negotiate understandings of, research evidence relative to other information, e.g. financial impact and local need, when considering reconfiguration.
2. At the meso level: how individual ‘champions’ and collective decision-making groups influence how evidence is used and consensus reached among providers and commissioners across health systems considering reconfiguration.

#### Case study 2: uptake of new national guidance in primary care to improve early diagnosis of suspected cancer

In 2015, NICE updated its clinical guidelines [22] for recognition and referral for suspected cancer which substantially lowered thresholds for investigation and shifted focus to signs and symptoms, to reflect how patients present in primary care. The guidance seeks to improve the quality and timeliness of diagnosis and, if implemented, is likely to significantly increase referrals. NICE has produced costing tools to help areas respond to potential demand increases and associated cost impacts [23]. Uptake may be influenced by general practitioners’ (GPs) responses to the guidelines [24] and the local context including, on the one hand, local activity by a wide range of organisations encouraging implementation while, on the other, potential pressure from hospitals and commissioners not to over-refer [25]. However, NICE also recommends that some investigations are now arranged in primary care (e.g. for colorectal cancer) when these previously required referral. This case will examine the following:

1. At the micro level: how general practitioners within primary care practices with historically lower and higher referral rates for suspected cancer have responded to new guidance, including profession-specific barriers and facilitators.
2. At the meso level: how involvement and interaction between different organisations, including clinical networks, commissioners, third sector, and service providers, have influenced responses to the referral guidance and its use to inform service planning.

#### Case study 3: using evidence to inform development of new virtual clinics within extended network of eye services

We will study innovations in outpatient services for treating chronic eye disease where demographic change is placing increasing pressure on hospitals. New service configurations are being developed by a number of providers within the NHS. The study will be conducted with a large specialist provider of ophthalmic services in England, which delivers 470,000 outpatient appointments per year through an organisational network (including partnerships with other providers) with multiple clinics across a large metropolitan area and the surrounding region. The Trust has piloted ‘virtual review’ clinics for stable glaucoma patients to improve resource use and enable provision closer to patients’ homes, although barriers to implementation were encountered [26].

A prospective study will analyse how evidence informs decision-making by the Trust and other providers within the network to move from the pilot phase to a wider implementation of virtual clinics across other sites, including responses to evidence for change by different sites and professional groups. This case will focus on the following:

1. At the micro level: types of evidence required by different professional groups, including clinical and managerial staff, to support roll-out of new clinics.
2. At the meso level: processes across the organisational network used to acquire, share, and interpret evidence to inform decision-making. This will include analysis of how counter evidence and other factors, e.g. perceived resource implications and local need, are negotiated and inform decision-making.

Case study data will be collected through semi-structured interviews, non-participant observation, and documentary analysis (Table 2). Interviews will be used to develop an account of the decision-making process from different stakeholder perspectives and obtain their views on barriers and enablers to the use of evidence as a tipping point for adopting innovation. Documentary analysis will map the types of evidence used at different stages of the decision-making process. For prospective studies (case studies 2 and 3), ethnographic methods, including non-participant observation of meetings and ‘shadowing’ key staff, will be used to trace decision-making processes prospectively (i.e. in ‘real time’) and examine how evidence is considered by decision-makers in both formal (e.g. planning meetings) and informal settings (e.g. corridor conversations). Documentary analysis will involve mapping the types of evidence used to inform decision-making and the ways in which it is consulted during different stages of decision-making processes, e.g. tracking references in meetings’ minutes. Interviews will be digitally recorded and professionally transcribed, and observational data will be recorded by the researchers in field journals.

**Table 2:** Data collection methods for case studies

Innovation case study	Interviews	Observations	Documentary analysis
1. Reconfiguring stroke services	Up to 25, including commissioners and providers of services in GM and other areas considering reconfiguration	Up to 20 h, including planning meetings at Trust level, commissioner and provider meetings, and other relevant decision-making authorities	Up to 100 documents, including meeting minutes, published research, grey literature, local data
2. National guidance for referral for suspected cancer	Up to 25 across two local health economies, including GP practices, clinical networks, and third sector	Up to 40 h, including commissioning meetings, GP training events, service planning meetings	Up to 50 documents, including guidelines, local service planning
3. New virtual clinics within extended network of eye services	Up to 25, including Trust board members, those leading innovation, and staff involved in implementation	Up to 40 h, including board meetings; innovation planning meetings, e.g. steering group; local planning in satellite sites; and shadowing key staff	Up to 50 documents, including meeting minutes, published research, grey literature, local data
Total	75 interviews	100 h	200 documents

The analysis of qualitative data from the case studies will combine inductive and deductive approaches [27], as thematic analysis will draw on ideas emerging from the empirical data as well as existing literature on evidence use in decision-making processes, including the role of contextual factors. Cross-case comparison will identify barriers and enablers of evidence use to support innovation at the micro and meso levels within the same setting and at the same level across different cases.

### Work stream 3: national survey and discrete choice experiment (DCE) to establish decision-makers’ preferences

Utilising the literature review, stakeholder feedback, and case study data, a survey of providers and commissioners will assess how preferences to introduce or diffuse innovations are influenced by characteristics of the evidence for change relative to other contextual factors (objective 3). The first part of the questionnaire will elicit preferences for different types and quality of evidence. The second part will be a DCE that will compare different decision-making groups’ preferences for the strength of evidence to examine tipping points in preferring one option over another.

We will develop a survey questionnaire for distribution to stakeholders (acute and primary care, providers, commissioners, managers, and clinicians). The questionnaire will have two parts. The first will contain questions about preferences for different types of evidence and quality of the evidence when judging whether or not to implement a new innovation. Types of evidence will include impact on health (mortality, quality of life); behaviour; knowledge; use of services; budget; and incremental cost-effectiveness. Quality of evidence will capture internal and external validities (e.g. extent to which evidence shows what it purports to show, extent to which findings are generalisable to the local area) and contextual factors (e.g. credibility of the person or organisation providing the evidence). We will ask respondents whether or not different types and quality of evidence are important and to rank them in order of importance. The different types and quality of evidence will be drawn from findings of the rapid review, focus groups, and case studies.

The second part will be a DCE to capture preferences of stakeholders for the strength of evidence needed (e.g. what the impact on health or the budget needs to be) to implement new innovations and how types of evidence are traded against one another.

The DCE will follow international best-practice guidelines [28] and be designed as follows:

1. We will identify types of evidence as described in the first part of the questionnaire. In the context of a DCE, these are referred to as attributes.
2. We will assign levels to these attributes (e.g. quantitative measurement of impact on health and use of services) based on real-world examples of innovations, derived from objectives 1 and 2 and systematic literature reviews.
3. We will design the DCE questionnaire using a pairwise choice framework. We will compile a set of pairwise scenarios that describe the feasible combinations of levels and attributes of new innovations. The number of pairwise choices will be reduced to a practical number for participants to answer.

The questionnaire will be piloted then administered to three groups of respondents: (1) acute care providers, (2) local commissioners, and (3) primary care providers (GPs and practice staff). Both managerial and clinical representatives will be sampled in the questionnaire.

Responses will be collected by online survey tools and hard-copy questionnaires delivered at face-to-face meetings. We will sample questionnaire respondents nationally and will use similar sampling methods to objective 1. We will also recruit respondents via newsletters distributed by Royal Colleges, professional organisations, and at professional conferences and meetings.

Sample size calculations for DCEs are not straightforward, depending on question format, complexity of choice tasks, desired precision of the results, degree of heterogeneity in the target population, availability of respondents, and need for subgroup analyses. A sample size of 300 is commonly recommended [29], which will be used here. We are not aware of any DCEs that have previously been conducted on this sample of respondents, but response rates achieved in previous DCEs with UK NHS staff have been around 55 % [30], and in health workers in high-income countries, they are around 50 % [31]. The same respondents will also complete the first part of the questionnaire, described above.

To analyse the data from the first part of the questionnaire, frequency tables will be created to describe whether or not different types and quality of evidence are important. We will cross-tabulate the responses by type of respondent and explore differences using chi-square tests. We will investigate differences in the ranking of different types and quality of evidence using rank-sum tests and Kendall’s coefficient of concordance.

The DCE data will be analysed using conditional logit regression analysis. The results will indicate which attribute (type of evidence) is most important to respondents and how this compares with the other attributes. We will explore the trade-offs the participants are willing to make between attributes, quantified using the marginal rates of substitution, which summarise how respondents trade-off values of the different attributes, e.g. what increase in total spending decision-makers are willing to trade-off for a 1 % reduction in mortality. We will model interaction effects, allowing us to investigate how preferences for one attribute vary dependent on another (e.g. impact of mortality on preferences at different levels of budget impact). We will also determine the predicted probability that a set of new innovations derived from different combinations of attributes would be selected based on the regression results. This will estimate the probability that innovations with particular values for the types of evidence will be selected and allows them to be ranked in terms of their order of preference.

### Work stream 4: developing guidance to improve evidence use

Combining findings from the literature review, focus groups, case studies, and DCE, factors to take into account when seeking, using, and producing evidence will be distilled into guidance for decision-makers and evaluators to inform decisions to introduce or adopt innovations. This may build on Lavis et al.’s five questions to inform strategies to improve the use of evidence by decision-makers (message, target audience, messenger, transfer processes, and evaluation) [32]. The guidance will describe the combinations of evidence (including type, strength, and presentation) needed to enable innovation, based on what is likely to satisfy different stakeholders in different contexts (e.g. in primary and acute care and innovation across single or multiple sites). A stakeholder workshop involving study participants will be held to gain feedback on the draft guidance as it is developed.

### Synthesis of quantitative and qualitative approaches

Our overall approach is to use mixed or ‘merged’ methods, which involves the integration of quantitative and qualitative methods [33]. This will be done in three ways. Firstly, the literature review will include both qualitative studies and descriptive statistics on evidence use, so that the relative influence of different forms of evidence on decision-making can be quantified, where such data exist. Secondly, findings from the stakeholder focus groups and case studies will inform the discrete choice experiment, as types of evidence use identified through the qualitative research will be included in the questionnaire and measured quantitatively. Thirdly, the dissemination of findings on evidence use will include both qualitative data on how different forms of evidence are used to inform decision-making as well as quantitative data on the contribution of different forms of information to decision-making in different contexts.

## Discussion

This study will enhance understanding of decision-makers’ use of diverse forms of evidence and the importance of contextual factors in shaping the ways in which evidence is used to inform decision-making on innovation. The findings will enhance understanding of how and why some evidence does inform decisions to introduce health care innovations and why barriers persist in other cases. It will also quantify decision-makers’ preferences, including the tipping point of evidence needed to shift stakeholders’ views. Guidance will be shared with key groups of decision-makers at different levels of the NHS, as well as evaluators, to inform how evidence is produced and used to satisfy a range of stakeholders when making decisions on adopting innovations. The study’s findings will improve understanding of how evidence can inform practice, including barriers and facilitators to its use. The study will produce generalisable knowledge on how to optimize the use of evidence to inform decisions on innovation adoption which, in turn, may help to accelerate and maximise the implementation of practices that improve patient outcomes.

The evidence needed to reach the tipping point for different groups will be determined using a DCE with providers and commissioners within both acute and primary care settings. A strength of this study is the use of quantitative and qualitative data to determine decision-makers’ preferences. The DCE will seek to measure the influence of diverse evidence and contextual factors, including professional interests and relationships, on decision-making. Thus, a practical challenge is translating insights from the qualitative data into appropriate options that can be used for eliciting participants’ preferences. For instance, if the qualitative data suggests that decision-making is a distributed process that unfolds over time, this might suggest the need to adapt the model of the lone, contemplative decision-maker typically used in a DCE (e.g. by administering the DCE to groups of participants rather than individuals).

The study’s findings will provide new insights into the ways in which evidence informs both the adoption of new innovations and the diffusion of existing innovations to new contexts, taking into account diverse types of evidence (e.g. academic research and local pilot data), different types of stakeholder, and contextual factors involved in decision-making. By combining qualitative and quantitative methods, the study will generate new knowledge regarding the types and quality of evidence that decision-makers prefer and the tipping point of evidence necessary to shift the perspectives of different groups of decision-makers. This includes groups or contexts that may typically be less receptive to change, e.g. medical professionals [34, 35], and where major system change is undertaken that involves multiple organisations and stakeholders [36].

### Ethics approval

This study was considered by the Chair of the UCL Research Ethics Committee on 29 February 2016 and is exempt from the requirement to obtain ethical approval.

## Abbreviations

CLAHRC: Collaborations for Leadership in Applied Health Research and Care
DCE: discrete choice experiment
GP: general practitioner
NHS: National Health Service
NICE: National Institute for Health and Care Excellence
